# Fine‐scale structure among mesophotic populations of the great star coral *Montastraea cavernosa* revealed by SNP genotyping

**DOI:** 10.1002/ece3.6340

**Published:** 2020-05-20

**Authors:** Crawford Drury, Rocío Pérez Portela, Xaymara M. Serrano, Marjorie Oleksiak, Andrew C. Baker

**Affiliations:** ^1^ Department of Marine Biology and Ecology Rosenstiel School of Marine and Atmospheric Science University of Miami Miami Florida; ^2^ Atlantic Oceanographic and Meteorological Laboratory National Oceanographic and Atmospheric Administration Miami Flordia; ^3^ Cooperative Institute for Marine and Atmospheric Studies University of Miami Miami Florida; ^4^ Present address: University of Barcelona Barcelona Spain; ^5^ Present address: Hawai'i Institute of Marine Biology University of Hawai'i at Mānoa Kāne'ohe Hawai'i

**Keywords:** Deep Reef Refugia, genetic connectivity, genotyping by sequencing, mesophotic reefs, *Montastraea cavernosa*

## Abstract

Mesophotic reefs (30‐150 m) have been proposed as potential refugia that facilitate the recovery of degraded shallow reefs following acute disturbances such as coral bleaching and disease. However, because of the technical difficulty of collecting samples, the connectivity of adjacent mesophotic reefs is relatively unknown compared with shallower counterparts. We used genotyping by sequencing to assess fine‐scale genetic structure of *Montastraea cavernosa* at two sites at Pulley Ridge, a mesophotic coral reef ecosystem in the Gulf of Mexico, and downstream sites along the Florida Reef Tract. We found differentiation between reefs at Pulley Ridge (~68 m) and corals at downstream upper mesophotic depths in the Dry Tortugas (28–36 m) and shallow reefs in the northern Florida Keys (Key Biscayne, ~5 m). The spatial endpoints of our study were distinct, with the Dry Tortugas as a genetic intermediate. Most striking were differences in population structure among northern and southern sites at Pulley Ridge that were separated by just 12km. Unique patterns of clonality and outlier loci allele frequency support these sites as different populations and suggest that the long‐distance horizontal connectivity typical of shallow‐water corals may not be typical for mesophotic systems in Florida and the Gulf of Mexico. We hypothesize that this may be due to the spawning of buoyant gametes, which commits propagules to the surface, resulting in greater dispersal and lower connectivity than typically found between nearby shallow sites. Differences in population structure over small spatial scales suggest that demographic constraints and/or environmental disturbances may be more variable in space and time on mesophotic reefs compared with their shallow‐water counterparts.

## Graphical abstract

Mesophotic reefs (30‐150 m) have been proposed as potential refugia that facilitate the recovery of degraded shallow reefs following acute disturbances at shallower depths, such as coral bleaching and disease. We find extensive population structure among mesophotic *Montastraea cavernosa*, even between nearby sites. Differences in population structure over small spatial scales suggest that demographic constraints and/or environmental disturbances may be more variable in space and time on mesophotic reefs compared with shallow‐water counterparts.

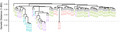

## 1 INTRODUCTION

Coral reefs are declining worldwide and face a future of increasingly frequent and severe stress. Overfishing, pollution, and changing climate are major drivers of coral decline (Hughes et al., 2017), but their impacts are not spatially homogenous (Van Hooidonk, Maynard, & Planes, 2013). Ecosystems that escape these and other stressors due to depth or remoteness may make an important contribution to the resilience and long‐term persistence of reefs (Gilmour, Smith, Heyward, Baird, & Pratchett, 2013).

Mesophotic coral reefs, typically defined as reefs at depths greater than ~30 m (Lesser, Slattery, & Leichter, 2009), are unique ecosystems that frequently occur adjacent to shallow reef communities where conspecifics may experience different impacts or stressor severity. This contrast led to the deep reef refugia hypothesis, which suggests that mesophotic reefs may provide refuge habitat and support healthy coral populations to facilitate the recovery of degraded shallow reefs via larval connectivity (Bongaerts, Ridgway, Sampayo, & Hoegh‐Guldberg, 2010; Gilmour et al., 2013; Glynn, 1996; Lesser et al., 2009). This hypothesis has since expanded to acknowledge that disturbance duration and return time are overlaid on functional, taxonomic, depth, and spatial factors that complicate its evaluation (Bongaerts & Smith, 2019). Some evidence suggests that declines associated with shallow reefs have been absent or less severe on their deeper counterparts (Bongaerts et al., 2010; Bridge, Hughes, Guinotte, & Bongaerts, 2013), but mesophotic reefs are also impaired by pollution, sedimentation, invasive species, thermal stress, disease, and storm damage (Robbart et al., 2009; Rocha et al., 2018; Smith et al., 2016; Smith, Holstein, & Ennis, 2019). Mesophotic reefs also have different algal symbiont communities, species composition, physiology, and community structure (Bongaerts et al., 2015; Goodbody‐Gringley, Marchini, Chequer, & Goffredo, 2015; Kahng et al., 2019; Lesser et al., 2010; Shlesinger, Grinblat, Rapuano, Amit, & Loya, 2018).

Mesophotic reefs may contribute to the recovery and persistence of their shallower counterparts primarily through larval subsidy over single or multiple generations (Holstein, Paris, Vaz, & Smith, 2016). Connectivity can be explored by using genetic markers to infer population structure, which serves as an indicator of the potential for migration across depths. Genetic data tightly correlate with biophysical migration rates in some coral populations (Matz, Treml, Aglyamova, & Bay, 2018), but has little explanatory power in others (Drury, Paris, Kourafalou, & Lirman, 2018), a difference which may be driven by taxonomic and demographic factors (e.g., asexual reproduction, population bottlenecks). Previous work using genetic data to infer connectivity and “reseeding potential” has shown that variable levels of horizontal connectivity between distant shallow reefs and vertical connectivity between reefs along a depth gradient can structure coral populations (Bongaerts et al., 2017; Eckert, Studivan, & Voss, 2019; Hammerman et al., 2017; Serrano et al., 2014, 2016; Studivan & Voss, 2018). The use of next‐generation sequencing data to infer migration has also resolved species‐specific vertical connectivity within Caribbean coral communities (Bongaerts et al., 2017; Hammerman et al., 2017), suggesting that the reseeding potential of mesophotic reefs may be important for some species assemblages, but is not universal.

*Montastraea cavernosa*, the Caribbean great star coral, is a suitable candidate for examining these patterns because it is capable of extensive vertical and horizontal connectivity (Nunes, Norris, & Knowlton, 2011). It is a gonochoric broadcast spawning coral which produces large eggs that may allow for longer larval duration (Szmant, 1991) and is also an extreme depth generalist, found between 0.5 and at least 95 m (Goreau & Wells, 1967). Previous work has demonstrated high horizontal connectivity between populations throughout the Caribbean, but these populations are not connected to reefs in West Africa or Brazil (Goodbody‐Gringley, Woollacott, & Giribet, 2012; Nunes, Norris, & Knowlton, 2009; Nunes et al., 2011). Vertical connectivity in this species appears to be site‐specific, resolved in some regions of Florida, the Gulf of Mexico, and the Cayman islands, but not in Bermuda, the U.S. Virgin Islands, Belize, or the Bahamas (Brazeau, Lesser, & Slattery, 2013; Eckert et al., 2019; Serrano et al., 2014; Studivan & Voss, 2018).

**FIGURE 1:**
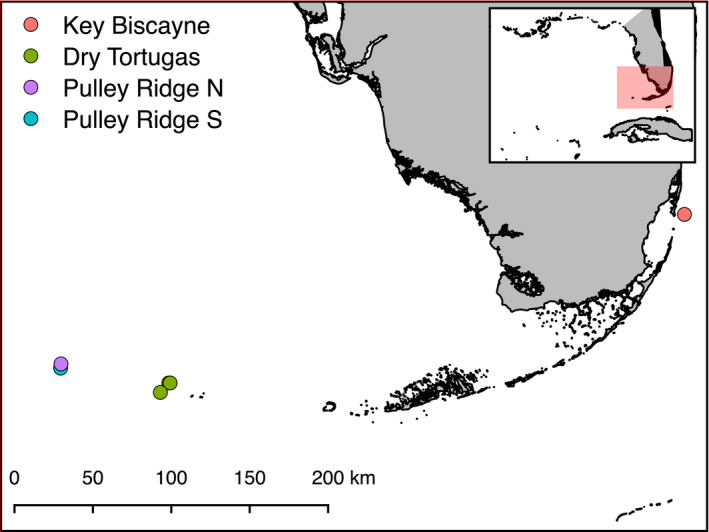
Sampling sites. Map of sampling sites along the Florida Reef Tract and Pulley Ridge. Points represent sites in Table 1, while colors represent populations distinguished by downstream analyses. Samples from Key Biscayne were collected at ~5 m depth, samples from the Dry Tortugas were collected from 28 to 36 m depth, and samples from Pulley Ridge were collected from ~68m depth

We take advantage of these life‐history traits, using genotyping by sequencing to examine population structure and connectivity in *M. cavernosa* samples from near the endpoints of the Florida Reef Tract (FRT) and Pulley Ridge (Figure 1). Pulley Ridge is a mesophotic reef in the Gulf of Mexico formed from drowned barrier islands on the West Florida Shelf at >60 m depth; it is considered the deepest known hermatypic reef in the United States (Jarrett et al., 2005). Pulley Ridge is within the Loop Current upstream of the Florida Reef Tract, creating the potential for export of pelagic larvae that are transported toward reefs (Sponaugle & Cowen, 2019) throughout *M. cavernosa's* depth range, representing a linkage between remote reefs and the Florida Reef Tract (Reed et al., 2019; Sponaugle & Cowen, 2019). We sampled shallow or mesophotic populations near the endpoints of the Florida Reef Tract to test the hypotheses that (a) there is unresolved population structure within the Florida Reef Tract between mesophotic and shallow coral populations and (b) the remote Pulley Ridge mesophotic ecosystem is part of the same population as other Florida corals.

## 2 MATERIALS AND METHODS

### 2.1 Collection, extraction, and library preparation

Corals were collected from Pulley Ridge in August 2013, the Dry Tortugas in August 2015, and Key Biscayne in September 2016 (Table 1, Figure 1). Samples came from two sites at ~68 m at Pulley Ridge (PR), three sites at depths of ~29 m in the Dry Tortugas (DT), and one site at a depth of 5 m at Key Biscayne (KB). Sites at Pulley Ridge were ~12 km apart on a large, subtle ridge with a cap of hermatypic coral fed by the Florida Loop Current (Jarrett et al., 2005). Samples were preserved in a chaotropic salt solution (“Chaos” buffer: 4.5 M guanidinium thiocyanate, 2% *N*‐lauroylsarcosine, 50 mM EDTA, 25 mM Tris‐HCL pH 7.5, 0.2% antifoam, 0.1 M beta‐mercaptoethanol) and transported to the University of Miami for processing. Samples were extracted using a silica column and vacuum manifold following Ivanova, Dewaard, and Hebert (2006). Extraction quality was verified on a 1% agarose gel before samples were quantified in triplicate (AccuBlue High‐Sensitivity dsDNA Quantitation Kit), and 50 ng of each sample was dried down in a 96‐well plate and rehydrated in 5 µl of water.

**TABLE 1:** Collections and depths

Region	*N* samples	*N* sites	Depth (m)
Key Biscayne (KB)	14	1	5 (shallow)
Dry tortugas (DT)	36	3	28 (upper mesophotic)
Pulley Ridge (PR)	21	2	67–68 (lower mesophotic)

Note

Collection locations by region, number of samples, sites, and depths. After initial clustering analysis, all 3 Dry Tortugas sites were assumed to be a single population and 2 Pulley Ridge sites were split by collection site, subsequently called “Pulley Ridge North” and “Pulley Ridge South.” Key Biscayne and Dry Tortugas samples are considered “Florida Reef Tract (FRT)” samples.

Libraries were prepared as in Drury et al. (2017) using a modified protocol of Elshire et al. (2011) and MseI (New England Biolabs) for digestion. An initial digestion with ApeKI produced inconsistent banding in different populations, likely due to the inhibition of the restriction enzyme. Samples were stored in a single batch of preservative and extracted in a single batch; thus, it is unlikely that preservation or extraction led to this population‐specific result. To resolve this, we instead used MseI to digest each library, producing consistent restriction fragment banding patterns across populations. Digests were bead‐purified at 0.5x bead concentration (0.1% Sera‐Mag beads, 20% PEG‐8000, 2.5 M NaCl) to remove small fragments. A 7‐9bp 5' barcode unique to each sample and a common adapter were ligated to each library, and postligation product was pooled and bead‐purified using 0.65x/1.0x double‐sided size selection to select fragments in the 100‐250 bp range. Pooled PCR samples were amplified for 18 cycles using primers complimentary to the oligonucleotides used in Illumina sequencing (Elshire et al., 2011), with an additional random 3bp overhang. The overhang decreases the proportion of low‐depth and singleton SNPs in the resulting reduced representation library, increasing the efficiency of downstream processing and facilitating analysis using the more frequent cutting restriction enzyme. PCR products were bead‐purified and visualized on a 1% agarose gel with a 100 bp DNA ladder to confirm size selection. Libraries were sequenced on an Illumina HiSeq 2500 with 75‐bp single‐end reads (Elim Biopharmaceuticals Inc.).

### 2.2 Data processing

We removed low‐quality bases (Phred < 20) at the leading and trailing end of reads, and reads where a 4‐bp sliding window average read quality fell below 20 as an initial filtration step using *Trimmomatic 0.32* (Bolger, Lohse, & Usadel, 2014). Reads were demultiplexed to sample using the *FastXToolkit* (http://hannonlab.cshl.edu/fastx_toolkit/). Alignment and SNP calling were performed using *bwa* (Li & Durbin, 2009) and *FreeBayes* (Garrison & Marth, 2012) within the *dDocent* pipeline using default settings (Puritz, Hollenbeck, & Gold, 2014) with the *Montastraea cavernosa* genome and no predefined population structure (ver. July 2018, http://matzlab.weebly.com/data‐‐code.html).

We created a combined *Symbiodiniaceae* reference following Manzello et al. (2019) from transcriptomes of all genera found in *M. cavernosa* (Goulet, Lucas, & Schizas, 2019): *Symbiodinium* (Bayer et al., 2012), *Breviolum* (http://sites.bu.edu/davieslab/data‐code/), *Cladocopium* (Davies, Marchetti, Ries, & Castillo, 2016), and *Durusdinium* (Ladner, Barshis, & Palumbi, 2012). We aligned raw reads using *bowtie2.3.5* with local settings, filtered to primary alignments with mapQ > 20, and quantified read count for each genus with *SAMtools 1.9*. This strategy is mutually exclusive between genera, meaning that a primary alignment to the *Cladocopium* portion of the combined reference indicates a read was a better fit than for any other genus. Approximately 0.25% of reads mapped to the *Symbiodiniaceae* references.

### 2.3 Analysis

The SNP matrix was filtered to include samples with at least 70% of loci called and loci in at least 92% of samples. To select only neutral markers, loci in LD (*R* ^2^ > 0.2) and loci not in HWE (*p* < .01) were identified with *VCFtools 0.1.13* (Danecek et al., 2011) and removed. We used *PCAdapt* (Luu, Bazin, & Blum, 2017) to separate loci potentially under selection (*n* = 182) for separate analysis, converting *p*‐values to *q*‐values and filtering at a 5% FDR. We then assessed clonality from hierarchical clustering of identity‐by‐state matrices generated by *SNPrelate* (Zheng et al., 2012) from the 13,123 remaining neutral loci. We chose 0.01 as a clonality threshold and removed all but one sample from each of the clonal groupings, yielding 64 samples in the subsequent analysis.

To examine structure, we used discriminant analysis in the *adegenet* package (Jombart, 2008) in R to cluster and visualize data using 21 principal components (~n/3) with populations determined by site as in Table 1 (KB *n* = 1 site, DT *n* = 3 sites, PR *n* = 2 sites). Strong overlap of all Dry Tortugas sites (Figure 3a) suggested populations defined as KB, DT, Pulley Ridge North, and Pulley Ridge South, which were used for downstream analyses (Tables 2 and 3 and Figures 3, 4, 5). We maintained separation between Pulley Ridge North and DT as populations in DAPC despite overlap due to the distance in hierarchical clustering and subsequent F‐statistics. We made pairwise comparisons of F_ST_ using *VCFtools* and used *ADMIXTURE 1.3* (Alexander & Lange, 2011) to infer individual ancestries for *K* values from 1 to 4 (minimum possible to maximum likely K based on DAPC), with the optimal K defined by the minimum cross‐validation error term (Alexander, Novembre, & Lange, 2009). There were small differences between *K* = 2 and *K* = 3, so we included all population numbers for comparison (Figure 4). We used the R package *vegan* (Oksanen et al., 2010) to test isolation by distance (9,999 permutations for significance) using the identity‐by‐state matrix described above. A 1,000‐bp window centered on the SNP position was extracted from the reference genome for 182 SNPs potentially under selection. These sequences were analyzed in *Blast2GO 5.2.5* (Conesa et al., 2005), compared with a custom blast database of cnidarians (taxid: 6,073). Allele frequencies of *F*_ST_ outlier loci were calculated with *VCFtools* and plotted, with minor alleles assigned based on global frequency. We compared depth and minor allele frequency with linear regression.

**TABLE 2:** Pairwise *F*_ST_ values

	KB	DT	PR‐N	PR‐S
KB				
DT	0.0136			
PR‐N	0.0291	0.0089		
PR‐S	0.0273	0.0045	0.0262	

Note

Pairwise *F*_ST_ values between populations below diagonal, calculated in VCFtools. Shading represents magnitude of *F*_ST_.

**TABLE 3:** Diversity and allelic frequency patterns

Population	*N*	Ng	Ng/*N*	Polymorphic sites	*H*_o_	*H*_e_	Na	Diversity (π)
KB	14	13	0.93	41.50%	0.082	0.081	1.081	0.0812
DT	36	36	1.00	60.70%	0.069	0.079	1.078	0.079
PR‐N	14	12	0.86	29.30%	0.055	0.071	1.07	0.0692
PR‐S	7	4	0.57	16.00%	0.064	0.062	1.063	0.063

Note

Diversity and allelic patterns for each population.

Nucleotide diversity = intrapopulation genetic diversity (Nei & Li, 1979).

The number of samples removed after clonality analysis is N‐Ng. All allele frequency calculations were made after clonal replicates were removed.

Abbreviations: H_E_, average expected heterozygosity; H_O_, average observed heterozygosity; *N*, sample size; NA, allelic richness, average number of alleles; Ng, number of genets; Ng/*N*, genet‐to‐ramet ratio; polymorphic sites, percentage of sites with multiple alleles within a population.

## 3 RESULTS

### 3.1 Data processing

The final SNP matrix contained 71 individuals and 13,123 neutral loci, with an additional 182 loci identified as outliers potentially under selection. Downstream analysis consisted of samples with an average of 92% of loci (range: 70%–100%) and loci called in an average of 97% of samples (range: 92%–100%). Average depth per call was 31.2 reads.

### 3.2 Population structure and allelic patterns

**FIGURE 2:**
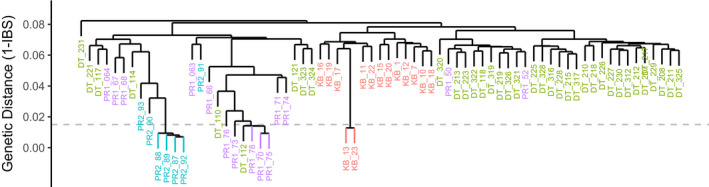
Hierarchical Clustering of Samples based on Identity by State (IBS). Hierarchical clustering of identity‐by‐state pairwise values for all samples. IBS was calculated from SNP matrix using *SNPrelate* and used to create a tree using the “complete” method. Samples are color‐coded by population as in Figure 1. Dashed horizontal line represents the chosen cutoff for clonality, and nodes below this value with samples from the same site were designated as nodes and are visualized with leaf color. All but one sample were randomly removed from each clonal grouping for downstream analysis

Hierarchical clustering of genetic distances based on identity by state (Figure 2) suggested the presence of clones. The genet‐to‐ramet ratio (N_g_/*N*) was highest in the Dry Tortugas, where we found no clones, and lowest at Pulley Ridge South, where 4 of 7 samples were from the same clonal group (Table 3 and Figure 2). Hierarchical clustering suggests population structure; the two Pulley Ridge sites are separated from the more closely related Key Biscayne and Dry Tortugas populations. All Key Biscayne samples clustered within a single node, maintaining separation from other populations. A subset of the Dry Tortugas populations clustered with each Pulley Ridge site, and Pulley Ridge assignments were also mixed. These mixed assignments closely corresponded to the admixture analysis for all values of *K*. We chose 0.01 as a clonality threshold and removed all but one sample from each of the clonal groupings, yielding 64 samples in the subsequent analysis.

**FIGURE 3:**
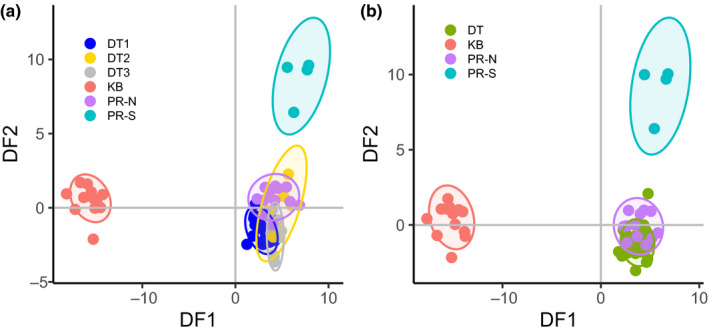
Discriminant Analysis of Principal components. Discriminant analysis was used to separate populations based on 13,132 neutral loci. (a) All sampling sites were considered as separate populations, producing large overlap within Dry Tortugas sites and Pulley Ridge North. (b) Populations defined based on all sites, but with Pulley Ridge North retained as a unique population due to hierarchical clustering and admixture results. Colors in panel (b) correspond to populations as shown in Figure 1, 2, and 5

*DAPC* resolved extensive overlap between the three Dry Tortugas sites (Figure 3a), and we considered these samples one population for subsequent analyses. This analysis separated the most distant Key Biscayne population along the first discriminant function and Pulley Ridge South along the second discriminant function (Figure 3b), while the Pulley Ridge North samples clustered with the Dry Tortugas population.

**FIGURE 4:**
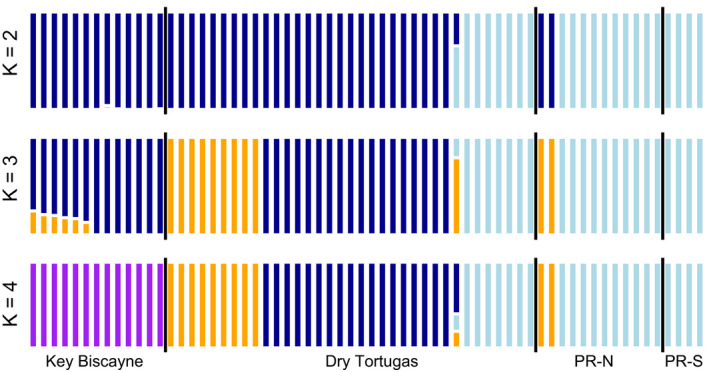
ADMIXTURE results. Results calculated from ADMIXTURE for population assignment at *K* = 2–4. *K* = 3 was the best grouping, selected based on the minimum cross‐validation error term. Colors represent ancestry assignment and are independent of colors in other figures

Admixture results indicated *K* = 2 was the optimal number of populations (Figure 4), where Pulley Ridge is a distinctive group. However, there was very little difference between *K* = 2 and *K* = 3 (CV error = 0.251 and 0.258, respectively). This assignment showed a third ancestral group in the Dry Tortugas in addition to samples with shared assignments with the other two regions. When examining *K* = 4 populations, assignments were virtually the same as *K* = 3; however, Key Biscayne is assigned to a distinct additional ancestral group. The separation of Dry Tortugas assignments did not relate to the three “sites” that were pooled based on initial DAPC analysis (Figure 3a); however, the results agreed almost exactly with hierarchical clustering. Six of nine Dry Tortugas samples with the primary Pulley Ridge ancestry assignment (*n* = 9, Figure 4) were found in the Pulley Ridge node of hierarchical clustering (Figure 2), and both Pulley Ridge samples with a Dry Tortugas ancestry assignment (*n* = 2, Figure 4) were found in the Dry Tortugas node (Figure 2). No outcomes from the admixture analysis supported separation between the two Pulley Ridge sites. Pairwise *F*_ST_ analysis supported the separation of Pulley Ridge sites in hierarchical clustering and DAPC results (Table 2), with the exception of low *F*_ST_ values between DT and PR. Pairwise *F*_ST_ supported the separation of Pulley Ridge sites as well as the distinctiveness of the Key Biscayne population. We found highly significant isolation by distance (*p* < .001), with 30% of variation explained by geographic distance (*R* ^2^ = 0.30).

Allelic richness (Na) was similar across all sites (Table 3), but Key Biscayne had the highest observed heterozygosity and nucleotide diversity. The Dry Tortugas had approximately equal allelic richness to Key Biscayne, despite the much larger Dry Tortugas sample size. Both Pulley Ridge sites had low heterozygosity, polymorphism, and nucleotide diversity.

### 3.3 Outlier loci

**FIGURE 5:**
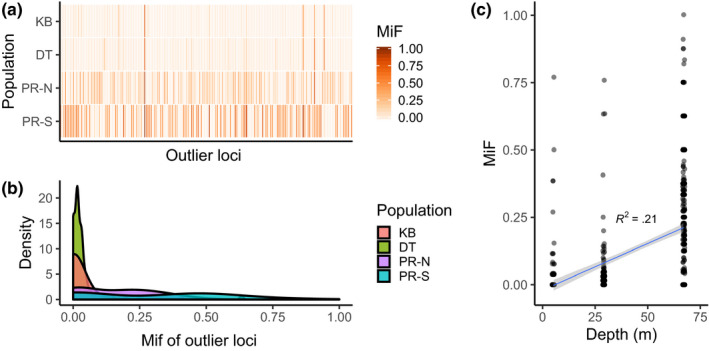
Patterns of Outlier Loci. (a) Comparison of minor allele frequency (MiF) across 182 outlier loci in each population, darker colors represent higher frequencies. (b) Density distribution of MiF for each population. For all comparisons, the minor allele was identified globally, so within‐population frequencies may be <0.5. (c) Linear fit of minor allele frequency by depth

The minor allele frequency (MiF, calculated from entire dataset) of the 182 outlier loci was highest within the Pulley Ridge region, where most loci had MiF > 0.25 (Figure 5a). Many outlier loci with high MiF at Pulley Ridge North are shared with Pulley Ridge South, but not with the Dry Tortugas or Key Biscayne. Pulley Ridge had a wider distribution of MiF among outlier loci (Figure 5b). There was a significant increase in minor allele frequency of outlier loci as depth increased (Figure 5c; linear regression *p* < .001, *R* ^2^ = 0.21). Of 182 outlier loci, 100 (54.9%) had blastx matches (*e*‐value < 0.001) and 62 has assigned gene ontologies (34.0%), most commonly involving ATP binding, G protein‐coupled receptor activity, ion binding, and various plasma membrane properties.

### 3.4 Symbiosis

Every sample in the dataset had reads that aligned to all four genera of *Symbiodiniaceae* (Figure S1). The proportion of reads aligning to *Cladocopium* was slightly higher in Pulley Ridge corals, which also had fewer reads aligning to *Symbiodinium*. Low proportions of reads study‐wide aligned to *Durusdinium*, except in one Dry Tortugas colony*.* Shallow and mesophotic reefs did not show substantial differences in proportion of reads aligning to different symbiont genera.

## 4 DISCUSSION

Connectivity is an important component of the health and resilience of contemporary reefs, and declining coral communities have prompted a focus on potential links between shallow and mesophotic populations. This study shows genetic structure distinguishing nearby mesophotic populations of *Montastraea cavernosa* at two sites (~68 m) from each other and from shallower downstream FRT populations. Moreover, these mesophotic populations appear to have variable horizontal connectivity with nearby mesophotic sites in the Dry Tortugas where there is infrequent but distinct signature of migration. Unique patterns of outlier loci and interindividual relatedness support differentiation among these populations.

Our findings indicate that Pulley Ridge and shallow FRT sites (Key Biscayne) are distinctive, but that Dry Tortugas sites may have intermediate connectivity with each of these two endpoints, generally agreeing with the conclusions of Serrano et al. (2014) who used microsatellites to demonstrate the presence, but not ubiquity, of genetic structure between shallow and mesophotic populations in this region. Our results also support the microsatellite‐based conclusions of Studivan and Voss (2018), who documented the isolation of Pulley Ridge from populations in Belize and the northwest Gulf of Mexico. However, our SNP‐based study also reveals evidence for fine‐scale genetic differentiation among the three mesophotic populations while also emphasizing isolation from downstream shallow reefs in the FRT. Serrano et al. (2014) found the Dry Tortugas had more vertical connectivity with local shallow populations than downstream populations in the Lower or Upper Keys. This pattern agrees with our hierarchical clustering and admixture analyses. Overall, there is little evidence for a completely discrete mesophotic population of *M. cavernosa* at the westernmost extent of the FRT (Dry Tortugas), at least at the depths sampled (28‐36 m). However, limited sampling and lack of paired shallow‐deep sampling locations (which minimize horizontal distance (Bongaerts et al., 2017; Hammerman et al., 2017; Serrano et al., 2014; Serrano et al., 2016; van Oppen, Bongaerts, Underwood, Peplow, & Cooper, 2011) hinder our ability to make strong conclusions. The use of the Key Biscayne as a shallow comparison may also introduce bias into our analysis because of horizontal distance and potential selective pressure associated with nearby coastal development. Shallow populations in the Dry Tortugas and Lower Keys may provide additional context, complementing the paired shallow‐deep sampling locations of other studies.

The genetic disconnect between Pulley Ridge and the Dry Tortugas also indicates the Pulley Ridge mesophotic reef ecosystem is moderately isolated. Hierarchical clustering, DAPC, and admixture analysis suggest they are distinct but indicate some shared ancestry and migration between these mesophotic populations. However, admixture analysis for any number of populations does not discriminate between the two Pulley Ridge sites, while DAPC and fixation indices show strong separation and hierarchical clustering, suggesting relatedness is much higher within than between Pulley Ridge sites. In this case, admixture analysis could be a more comprehensive assessment of structure (i.e., not focused on heterozygosity‐based metrics or maximizing discriminant functions); however, the interaction of demography and small sample sizes precludes us from rarefying our results and makes it difficult to determine whether this and other metrics are biased, even after clonal replicates were removed. Bayesian methods such as STRUCTURE and ADMIXTURE are generally restricted to larger sample sizes when using microsatellites (Porras‐Hurtado et al., 2013), but because SNPs are biallelic, as few as four samples are required to obtain accurate allele frequencies (Shi et al., 2010). Conversely, outlier loci suggest that Pulley Ridge is discrete from both FRT sites and there is some differentiation between northern and southern Pulley Ridge sites. The low genetic diversity and polymorphism of both Pulley Ridge sites likely contribute to these analyses and may be reflective of inbreeding or a selective event.

The patterns described here could also be influenced by clonality within sites. No biological replicates were collected in this study, so direct assessment of intraindividual variation using a distance threshold (following (Drury, Greer, Baums, Gintert, & Lirman, 2019; Manzello et al., 2019) was not possible. Traditionally, many studies have assumed that *M. cavernosa* populations reproduced primarily sexually (Szmant, 1991), but more recent work has documented common asexual reproduction in massive corals (Manzello et al., 2019), likely due to physical disturbance (Foster et al., 2013). There is some evidence for potential internal fertilization in *M. cavernosa* (Hagman, Gittings, & Vize, 1998), but as a gonochoric species there is no clear mechanism for creating genetically identical colonies other than parthenogenesis (Combosch & Vollmer, 2013), which is unknown in *M. cavernosa* but could be important for reproductive assurance in an isolated population (Yund, 2000). *Montastraea cavernosa* exhibits a massive or plating morphology (Lesser et al., 2010), and while the physical impacts of hurricanes are less significant at depth (Smith et al., 2019), evidence of storm damage has been documented in plating corals > 50m depth (Bongaerts, Muir, Englebert, Bridge, & Hoegh‐Guldberg, 2013; Woodley et al., 1981). These impacts suggest that asexual reproduction due to physical fragmentation is possible, but we expect these populations to be mostly nonclonal based on the general rarity of clones as assessed by microsatellites (Eckert et al., 2019; Serrano et al., 2014). Conversely, our results agree with the high clonality documented by Studivan and Voss (2018) at these same sites, confirming that clonal reproduction is an important component of Pulley Ridge coral communities and providing an example of robust conclusions reached by both microsatellites and SNPs. Small sample sizes and low diversity may have a particularly strong influence on comparisons between Pulley Ridge sites, but the logistical limitations of collecting mesophotic corals limit our analysis and preclude us from re‐analyzing data without closely related individuals other than the clonal replicates removed here.

Population differentiation among *M. cavernosa* from nearby mesophotic reefs appears to be highly site‐specific. For example, Studivan and Voss (2018) documented variable horizontal connectivity between nearby mesophotic reefs in the northwest Gulf of Mexico, while Eckert et al. (2019) found high horizontal connectivity among mesophotic sites in Belize. Not surprisingly, larger geographic distances between mesophotic sites tend to encourage population differentiation (Studivan & Voss, 2018), and this study supports earlier suggestions that horizontal connectivity among mesophotic sites may be more complex and variable than connectivity among shallow sites (Serrano et al., 2014). We hypothesize this is a consequence of the life‐history strategy of this species, in which sperm and eggs are synchronously released from male and female colonies and buoyant fertilized eggs (Wyers, Barnes, & Smith, 1991) spend their early development at the surface (Hagman, Gittings, & Deslarzes, 1998). These larvae may be subject to longer time to the surface, contributing to horizontal dispersal prior to the onset of larval motility and settlement at depth. If this is the case, the commitment of these propagules to spending early ontogeny in shallow depths could result in greater population differentiation over smaller spatial scales compared with their shallow counterparts. Biophysical simulations of this process in *Orbicella faveolata* in the USVI suggest that most propagules are transported to shallower settlement habitat, but a substantial number of propagules from the ~30‐50 m depth also settle in that range (Holstein et al., 2016). However, the buoyancy of gametes varies between species and is a critical determinant of this pattern.

The Pulley Ridge ecosystem is approximately 60 km to the west of the Dry Tortugas (Jarrett et al., 2005), which is the westernmost extent of the FRT. This region may be more heavily influenced by oceanographic conditions including the Loop Current, which not only drives circulation in the eastern Gulf of Mexico (Hurlburt & Thompson, 1980; Kourafalou & Kang, 2012), but also produces eddies which stochastically influence larval connectivity in the Florida Keys (Sponaugle, Lee, Kourafalou, & Pinkard, 2005) and exacerbate the highly variable nature of larval connectivity (Hedgecock & Pudovkin, 2011). Biophysical modeling of coral reef fish larvae shows that transport can occur between Pulley Ridge and the Dry Tortugas in as little as 7 days (Vaz et al., 2016), which is likely within the larval competency period of *M. cavernosa* (Nunes et al., 2011). We found equivocal evidence (disagreement between ADMIXTURE and *F*_ST_ outcomes) that this process is actually occurring in *M. cavernosa*. Results from simulated migration patterns also indicate common horizontal asymmetry, with more frequent “migration” in the downstream (east and north) direction of the Florida Current except in the Lower Keys, where westward transport is also common due to the complexity of oceanographic patterns in this area (Kourafalou & Kang, 2012; Staaterman, Paris, & Helgers, 2012). Settlement and juvenile survivorship are also critical components of connected populations (Harrison & Wallace, 1990), and if the Pulley Ridge ecosystem is highly distinctive, a phenotype–environment mismatch (Marshall, Monro, Bode, Keough, & Swearer, 2010) could be occurring for propagules transported to the Florida Reef Tract. Although we consider both Dry Tortugas (~30 m) and Pulley Ridge (~70 m) corals to be mesophotic, they are at substantially different depths.

Outlier loci in this study may reflect demographic effects or selection, while our small sample sizes at Pulley Ridge are disadvantageous, it is unlikely that such large effect sizes are purely due to sampling of nonclonal individuals. Both Pulley Ridge sites show unique patterns of outlier loci, with high minor allele frequencies compared with Key Biscayne and the Dry Tortugas. While population structure (demography) may be driving these differences, distance‐based metrics explain only ~30% of variation. The minor allele frequency of these loci increases with depth, similar to the findings of Bongaerts et al. (2017), explaining 21% of the variance and suggesting that adaptation to environmental conditions plays some role in addition to demography. Furthermore, mesophotic conditions require adaptation and plasticity of the whole coral holobiont to adjust to drastically different conditions, so some adaptive differences should be expected. Differences between environmental conditions including light, water chemistry, and plankton density (Lesser et al., 2009) lead to changes in feeding rates, morphological differences, photophysiology, and symbiont communities (Bongaerts et al., 2015; Goodbody‐Gringley et al., 2015; Lesser et al., 2010; Polinski & Voss, 2018), which could represent selective pressure on mesophotic reefs.

We also document diverse algal symbionts, finding reads aligning to all four major genera of *Symbiodiniaceae* from Caribbean scleractinian corals in every sample. Interestingly, Serrano et al. (2014) found that *M. cavernosa* from Florida harbored mostly *Cladocopium*, with a few examples of *Durusdinium* and *Breviolum*, mostly at shallower sites. Caribbean‐wide, *Cladocopium* tends to be the most common symbiont in mesophotic *M. cavernosa,* and no regions have previously documented all four genera (Goulet et al., 2019). The consistent diversity of this community across depths also contrasts with previous results that suggest depth‐generalist species that harbor multiple symbiont genera exhibit depth‐related partitioning of relative frequencies (reviewed in Ref. (Kahng et al., 2019)). Our analysis cannot precisely determine relative abundance because of variation in reference size (47–99 Mbp), but this study demonstrates the utility of host‐focused sequencing data for broad descriptions of symbiosis that may capture more variation than other methods (e.g., DGGE, targeted qPCR).

Vertical and horizontal connectivity may be important for reef recovery, but the contributions of mesophotic ecosystems to shallow reef populations appear to be highly site and species‐specific (Bongaerts et al., 2017). Here, we report considerable population structure within adjacent mesophotic systems and across the Florida Reef Tract, highly clonality at Pulley Ridge, and the presence of diverse symbiont assemblages in both shallow and mesophotic *M. cavernosa*. We demonstrate that the endpoints of the Florida Reef Tract harbor *M. cavernosa* communities which are genetically distinct, potentially due to long horizontal distances and differences in habitat or environment between shallow and mesophotic reefs. In addition, we find that Pulley Ridge coral communities are isolated and low diversity, with potential selective pressure not found at nearby mesophotic reefs. These trends could be reflective of local adaptation to unique circumstances that are uncommon on other mesophotic reefs, or may reflect a recent mortality or bottleneck event. The role of clonality is also unclear, but suggests the importance of founder effects in these isolated, mesophotic systems. Future work should continue to focus on paired populations and upstream communities which may contribute to this area via larval seeding, better explaining the potential contributions of mesophotic reefs to contemporary reef resilience.

## CONFLICT OF INTEREST

The authors declare no conflict of interest.

## AUTHOR CONTRIBUTION

**Crawford Drury:** Data curation (lead); Formal analysis (lead); Investigation (lead); Visualization (lead); Writing‐original draft (lead); Writing‐review & editing (lead). **Rocío Pérez Portela:** Data curation (supporting); Methodology (supporting); Writing‐review & editing (supporting). **Xaymara M. Serrano:** Formal analysis (supporting); Visualization (supporting); Writing‐review & editing (supporting). **Marjorie Oleksiak:** Conceptualization (equal); Funding acquisition (equal); Writing‐original draft (supporting); Writing‐review & editing (supporting). **Andrew C. Baker:** Conceptualization (equal); Funding acquisition (equal); Writing‐original draft (supporting); Writing‐review & editing (supporting).

## Supporting information

Fig S1

## Data Availability

The data that support the findings of this study are openly available at Dryad https://doi.org/10.5061/dryad.0rxwdbrvv. Raw reads are deposited at NCBI SRA under PRJNA611636 (Drury, Perez Portela, Serrano, Oleksiak, & Baker, 2019).

## References

[ece36340-bib-0001] Alexander, D. H. , & Lange, K. (2011). Enhancements to the ADMIXTURE algorithm for individual ancestry estimation. BMC Bioinformatics, 12(1), 246 10.1186/1471-2105-12-246 21682921 PMC3146885

[ece36340-bib-0002] Alexander, D. H. , Novembre, J. , & Lange, K. (2009). Fast model‐based estimation of ancestry in unrelated individuals. Genome Research, 19(9), 1655–1664. 10.1101/gr.094052.109 19648217 PMC2752134

[ece36340-bib-0003] Bayer, T. , Aranda, M. , Sunagawa, S. , Yum, L. K. , DeSalvo, M. K. , Lindquist, E. , … Medina, M. (2012). Symbiodinium transcriptomes: Genome insights into the dinoflagellate symbionts of reef‐building corals. PLoS ONE, 7(4), e35269 10.1371/journal.pone.0035269 22529998 PMC3329448

[ece36340-bib-0004] Bolger, A. M. , Lohse, M. , & Usadel, B. (2014). Trimmomatic: A flexible trimmer for Illumina sequence data. Bioinformatics, 30(15), 2114–2120. 10.1093/bioinformatics/btu170 24695404 PMC4103590

[ece36340-bib-0005] Bongaerts, P. , Frade, P. R. , Hay, K. B. , Englebert, N. , Latijnhouwers, K. R. W. , Bak, R. P. M. , … Hoegh‐Guldberg, O. (2015). Deep down on a Caribbean reef: Lower mesophotic depths harbor a specialized coral‐endosymbiont community. Scientific Reports, 5(1), 7652 10.1038/srep07652 25564461 PMC4285725

[ece36340-bib-0006] Bongaerts, P. , Muir, P. , Englebert, N. , Bridge, T. , & Hoegh‐Guldberg, O. (2013). Cyclone damage at mesophotic depths on Myrmidon Reef (GBR). Coral Reefs, 32(4), 935–935. 10.1007/s00338-013-1052-y

[ece36340-bib-0007] Bongaerts, P. , Ridgway, T. , Sampayo, E. , & Hoegh‐Guldberg, O. (2010). Assessing the ‘deep reef refugia’ hypothesis: Focus on Caribbean reefs. Coral Reefs, 29(2), 309–327. 10.1007/s00338-009-0581-x

[ece36340-bib-0008] Bongaerts, P. , Riginos, C. , Brunner, R. , Englebert, N. , Smith, S. R. , & Hoegh‐Guldberg, O. (2017). Deep reefs are not universal refuges: Reseeding potential varies among coral species. Science Advances, 3(2), e1602373 10.1126/sciadv.1602373 28246645 PMC5310828

[ece36340-bib-0009] Bongaerts, P. , & Smith, T. B. (2019). Beyond the “Deep Reef Refuge” hypothesis: A conceptual framework to characterize persistence at depth In Loya Y., Puglise K. A., & Bridge T. C. L. (Eds.), Mesophotic coral ecosystems (pp. 881–895). Berlin, Germany: Springer.

[ece36340-bib-0010] Brazeau, D. A. , Lesser, M. P. , & Slattery, M. (2013). Genetic structure in the coral, *Montastraea cavernosa*: Assessing genetic differentiation among and within mesophotic reefs. PLoS ONE, 8(5), e65845 10.1371/journal.pone.0065845 23734263 PMC3666989

[ece36340-bib-0011] Bridge, T. C. , Hughes, T. P. , Guinotte, J. M. , & Bongaerts, P. (2013). Call to protect all coral reefs. Nature Climate Change, 3(6), 528–530. 10.1038/nclimate1879

[ece36340-bib-0012] Combosch, D. J. , & Vollmer, S. V. (2013). Mixed asexual and sexual reproduction in the Indo‐Pacific reef coral *Pocillopora damicornis* . Ecology and Evolution, 3(10), 3379–3387. 24223276 10.1002/ece3.721 PMC3797485

[ece36340-bib-0013] Conesa, A. , Götz, S. , García‐Gómez, J. M. , Terol, J. , Talón, M. , & Robles, M. (2005). Blast2GO: A universal tool for annotation, visualization and analysis in functional genomics research. Bioinformatics, 21(18), 3674–3676. 10.1093/bioinformatics/bti610 16081474

[ece36340-bib-0014] Danecek, P. , Auton, A. , Abecasis, G. , Albers, C. A. , Banks, E. , DePristo, M. A. , … Durbin, R. (2011). The variant call format and VCFtools. Bioinformatics, 27(15), 2156–2158. 10.1093/bioinformatics/btr330 21653522 PMC3137218

[ece36340-bib-0015] Davies, S. W. , Marchetti, A. , Ries, J. B. , & Castillo, K. D. (2016). Thermal and pCO2 stress elicit divergent transcriptomic responses in a resilient coral. Frontiers in Marine Science, 3, 112 10.3389/fmars.2016.00112

[ece36340-bib-0016] Drury, C. , Greer, J. B. , Baums, I. , Gintert, B. , & Lirman, D. (2019). Clonal diversity impacts coral cover in *Acropora cervicornis* thickets: Potential relationships between density, growth, and polymorphisms. Ecology and Evolution, 9(8), 4518–4531. 31031924 10.1002/ece3.5035 PMC6476746

[ece36340-bib-0017] Drury, C. , Paris, C. B. , Kourafalou, V. H. , & Lirman, D. (2018). Dispersal capacity and genetic relatedness in *Acropora cervicornis* on the Florida Reef Tract. Coral Reefs, 37(2), 585–596. 10.1007/s00338-018-1683-0

[ece36340-bib-0018] Drury, C. , Perez Portela, R. , Serrano, X. , Oleksiak, M. , & Baker, A. C. (2019). Data supporting “Fine‐scale structure among mesophotic populations of the great star coral *Montastraea cavernosa* revealed by SNP genotyping. Dryad; doi: 10.5061/dryad.0rxwdbrvv PMC7319168 32607208

[ece36340-bib-0019] Drury, C. , Schopmeyer, S. , Goergen, E. , Bartels, E. , Nedimyer, K. , Johnson, M. , … Lirman, D. (2017). Genomic patterns in *Acropora cervicornis* show extensive population structure and variable genetic diversity. Ecology and Evolution, 7(16), 6188–6200. 10.1002/ece3.3184 28861224 PMC5574808

[ece36340-bib-0020] Eckert, R. J. , Studivan, M. S. , & Voss, J. D. (2019). Populations of the coral species *Montastraea cavernosa* on the Belize Barrier Reef lack vertical connectivity. Scientific Reports, 9(1), 1–11. 10.1038/s41598-019-43479-x 31076586 PMC6510931

[ece36340-bib-0021] Elshire, R. J. , Glaubitz, J. C. , Sun, Q. , Poland, J. A. , Kawamoto, K. , Buckler, E. S. , & Mitchell, S. E. (2011). A robust, simple genotyping‐by‐sequencing (GBS) approach for high diversity species. PLoS ONE, 6(5), e19379 10.1371/journal.pone.0019379 21573248 PMC3087801

[ece36340-bib-0022] Foster, N. L. , Baums, I. B. , Sanchez, J. A. , Paris, C. B. , Chollett, I. , Agudelo, C. L. , … Mumby, P. J. (2013). Hurricane‐driven patterns of clonality in an ecosystem engineer: The Caribbean coral *Montastraea annularis* . PLoS ONE, 8(1), e53283 10.1371/journal.pone.0053283 23308185 PMC3538762

[ece36340-bib-0023] Garrison, E. , & Marth, G. (2012). Haplotype‐based variant detection from short‐read sequencing. Arxiv, 1‐9. https://arxiv.org/pdf/1207.3907.pdf

[ece36340-bib-0024] Gilmour, J. P. , Smith, L. D. , Heyward, A. J. , Baird, A. H. , & Pratchett, M. S. (2013). Recovery of an isolated coral reef system following severe disturbance. Science, 340(6128), 69–71. 10.1126/science.1232310 23559247

[ece36340-bib-0025] Glynn, P. W. (1996). Coral reef bleaching: Facts, hypotheses and implications. Global Change Biology, 2(6), 495–509. 10.1111/j.1365-2486.1996.tb00063.x

[ece36340-bib-0026] Goodbody‐Gringley, G. , Marchini, C. , Chequer, A. D. , & Goffredo, S. (2015). Population structure of *Montastraea cavernosa* on shallow versus mesophotic reefs in Bermuda. PLoS ONE, 10(11), e0142427 10.1371/journal.pone.0142427 26544963 PMC4636301

[ece36340-bib-0027] Goodbody‐Gringley, G. , Woollacott, R. M. , & Giribet, G. (2012). Population structure and connectivity in the Atlantic scleractinian coral *Montastraea cavernosa* (Linnaeus, 1767). Marine Ecology, 33(1), 32–48. 10.1111/j.1439-0485.2011.00452.x

[ece36340-bib-0028] Goreau, T. F. , & Wells, J. (1967). The shallow‐water Scleractinia of Jamaica: Revised list of species and their vertical distribution range. Bulletin of Marine Science, 17(2), 442–453.

[ece36340-bib-0029] Goulet, T. L. , Lucas, M. Q. , & Schizas, N. V. (2019). Symbiodiniaceae genetic diversity and symbioses with hosts from shallow to mesophotic coral ecosystems In Loya Y., Puglise K. A., & Bridge T. C. L. (Eds.), Mesophotic Coral Ecosystems (pp. 537–551). Berlin, Germany: Springer.

[ece36340-bib-0030] Hagman, D. K. , Gittings, S. R. , & Deslarzes, K. J. (1998). Timing, species participation, and environmental factors influencing annual mass spawning at the Flower Garden Banks (Northwest Gulf of Mexico). Gulf of Mexico Science, 16(2), 170–179. 10.18785/goms.1602.06

[ece36340-bib-0031] Hagman, D. K. , Gittings, S. R. , & Vize, P. D. (1998). Fertilization in broadcast‐spawning corals of the Flower Garden Banks National Marine Sanctuary. Gulf of Mexico Science, 16(2), 7 10.18785/goms.1602.07

[ece36340-bib-0032] Hammerman, N. M. , Rivera‐Vicens, R. E. , Galaska, M. P. , Weil, E. , Appledoorn, R. S. , Alfaro, M. , & Schizas, N. V. (2017). Population connectivity of the plating coral *Agaricia lamarcki* from southwest Puerto Rico. Coral Reefs, 37, 183–191.

[ece36340-bib-0033] Harrison, P. , & Wallace, C. (1990). Reproduction, dispersal and recruitment of scleractinian corals. Ecosystems of the World, 25, 133–207.

[ece36340-bib-0034] Hedgecock, D. , & Pudovkin, A. I. (2011). Sweepstakes reproductive success in highly fecund marine fish and shellfish: A review and commentary. Bulletin of Marine Science, 87(4), 971–1002. 10.5343/bms.2010.1051

[ece36340-bib-0035] Holstein, D. M. , Paris, C. B. , Vaz, A. C. , & Smith, T. B. (2016). Modeling vertical coral connectivity and mesophotic refugia. Coral Reefs, 35(1), 23–37. 10.1007/s00338-015-1339-2

[ece36340-bib-0036] Hughes, T. P. , Barnes, M. L. , Bellwood, D. R. , Cinner, J. E. , Cumming, G. S. , Jackson, J. B. , … Morrison, T. H. (2017). Coral reefs in the Anthropocene. Nature, 546(7656), 82–90. 28569801 10.1038/nature22901

[ece36340-bib-0037] Hurlburt, H. , & Thompson, J. D. (1980). A numerical study of Loop Current intrusions and eddy shedding. Journal of Physical Oceanography, 10(10), 1611–1651. 10.1175/1520-0485(1980)010<1611:ANSOLC>2.0.CO;2

[ece36340-bib-0038] Ivanova, N. V. , Dewaard, J. R. , & Hebert, P. D. N. (2006). An inexpensive, automation‐friendly protocol for recovering high‐quality DNA. Molecular Ecology Notes, 6(4), 998–1002. 10.1111/j.1471-8286.2006.01428.x

[ece36340-bib-0039] Jarrett, B. D. , Hine, A. C. , Halley, R. B. , Naar, D. F. , Locker, S. D. , Neumann, A. C. , … Ciembronowicz, K. (2005). Strange bedfellows—a deep‐water hermatypic coral reef superimposed on a drowned barrier island; southern Pulley Ridge, SW Florida platform margin. Marine Geology, 214(4), 295–307. 10.1016/j.margeo.2004.11.012

[ece36340-bib-0040] Jombart, T. (2008). adegenet: A R package for the multivariate analysis of genetic markers. Bioinformatics, 24(11), 1403–1405. 10.1093/bioinformatics/btn129 18397895

[ece36340-bib-0041] Kahng, S. E. , Akkaynak, D. , Shlesinger, T. , Hochberg, E. J. , Wiedenmann, J. , Tamir, R. , & Tchernov, D. (2019). Light, temperature, photosynthesis, heterotrophy, and the lower depth limits of mesophotic coral ecosystems In Loya Y., Puglise K. A., & Bridge T. C. L. (Eds.), Mesophotic coral ecosystems (pp. 801–828). Berlin, Germany: Springer.

[ece36340-bib-0042] Kourafalou, V. H. , & Kang, H. (2012). Florida Current meandering and evolution of cyclonic eddies along the Florida Keys Reef Tract: Are they interconnected? Journal of Geophysical Research: Oceans, 117(C5), 1–25. 10.1029/2011JC007383

[ece36340-bib-0043] Ladner, J. T. , Barshis, D. J. , & Palumbi, S. R. (2012). Protein evolution in two co‐occurring types of Symbiodinium: An exploration into the genetic basis of thermal tolerance in Symbiodinium clade D. BMC Evolutionary Biology, 12(1), 217 10.1186/1471-2148-12-217 23145489 PMC3740780

[ece36340-bib-0044] Lesser, M. P. , Slattery, M. , & Leichter, J. J. (2009). Ecology of mesophotic coral reefs. Journal of Experimental Marine Biology and Ecology, 375(1), 1–8. 10.1016/j.jembe.2009.05.009

[ece36340-bib-0045] Lesser, M. P. , Slattery, M. , Stat, M. , Ojimi, M. , Gates, R. D. , & Grottoli, A. (2010). Photoacclimatization by the coral *Montastraea cavernosa* in the mesophotic zone: Light, food, and genetics. Ecology, 91(4), 990–1003. 10.1890/09-0313.1 20462114

[ece36340-bib-0046] Li, H. , & Durbin, R. (2009). Fast and accurate short read alignment with Burrows‐Wheeler transform. Bioinformatics, 25(14), 1754–1760. 10.1093/bioinformatics/btp324 19451168 PMC2705234

[ece36340-bib-0047] Luu, K. , Bazin, E. , & Blum, M. G. (2017). pcadapt: An R package to perform genome scans for selection based on principal component analysis. Molecular Ecology Resources, 17(1), 67–77. 27601374 10.1111/1755-0998.12592

[ece36340-bib-0048] Manzello, D. P. , Matz, M. V. , Enochs, I. C. , Valentino, L. , Carlton, R. D. , Kolodziej, G. , … Jankulak, M. (2019). Role of host genetics and heat tolerant algal symbionts in sustaining populations of the endangered coral *Orbicella faveolata* in the Florida Keys with ocean warming. Global Change Biology, 25(3), 1016–1031. 30552831 10.1111/gcb.14545

[ece36340-bib-0049] Marshall, D. , Monro, K. , Bode, M. , Keough, M. , & Swearer, S. (2010). Phenotype–environment mismatches reduce connectivity in the sea. Ecology Letters, 13(1), 128–140. 19968695 10.1111/j.1461-0248.2009.01408.x

[ece36340-bib-0050] Matz, M. V. , Treml, E. A. , Aglyamova, G. V. , & Bay, L. K. (2018). Potential and limits for rapid genetic adaptation to warming in a Great Barrier Reef coral. PLoS Genetics, 14(4), e1007220 10.1371/journal.pgen.1007220 29672529 PMC5908067

[ece36340-bib-0051] Nei, M. , & Li, W. H. (1979). Mathematical model for studying genetic variation in terms of restriction endonucleases. Proceedings of the National Academy of Sciences, 76(10), 5269–5273. 10.1073/pnas.76.10.5269 PMC413122 291943

[ece36340-bib-0052] Nunes, F. , Norris, R. , & Knowlton, N. (2009). Implications of isolation and low genetic diversity in peripheral populations of an amphi‐Atlantic coral. Molecular Ecology, 18(20), 4283–4297. 10.1111/j.1365-294X.2009.04347.x 19765228

[ece36340-bib-0053] Nunes, F. L. , Norris, R. D. , & Knowlton, N. (2011). Long distance dispersal and connectivity in amphi‐Atlantic corals at regional and basin scales. PLoS ONE, 6(7), e22298 10.1371/journal.pone.0022298 21799816 PMC3142122

[ece36340-bib-0054] Oksanen, J. , Blanchet, F. G. , Kindt, R. , Legendre, P. , O'hara, R. , Simpson, G. L. , … Wagner, H. (2010). vegan: Community ecology package. R package version 1.17‐2 In R Development Core Team (Ed.), R: A language and environment for statistical computing. Vienna, Austria: R Foundation for Statistical Computing.

[ece36340-bib-0055] Polinski, J. M. , & Voss, J. D. (2018). Evidence of photoacclimatization at mesophotic depths in the coral‐Symbiodinium symbiosis at Flower Garden Banks National Marine Sanctuary and McGrail Bank. Coral Reefs, 37(3), 779–789. 10.1007/s00338-018-1701-2

[ece36340-bib-0056] Porras‐Hurtado, L. , Ruiz, Y. , Santos, C. , Phillips, C. , Carracedo, Á. , & Lareu, M. (2013). An overview of STRUCTURE: Applications, parameter settings, and supporting software. Frontiers in Genetics, 4, 98 10.3389/fgene.2013.00098 23755071 PMC3665925

[ece36340-bib-0057] Puritz, J. B. , Hollenbeck, C. M. , & Gold, J. R. (2014). dDocent: A RADseq, variant‐calling pipeline designed for population genomics of non‐model organisms. PeerJ, 2, e431. 24949246 10.7717/peerj.431 PMC4060032

[ece36340-bib-0058] Reed, J. K. , Farrington, S. , David, A. , Harter, S. , Pomponi, S. A. , Diaz, M. C. , … Kourafalou, V. H. (2019). Pulley ridge, gulf of Mexico, USA In In Loya Y., Puglise K. A., & Bridge T. C. L. (Eds.), Mesophotic coral ecosystems (pp. 57–69). Berlin, Germany: Springer.

[ece36340-bib-0059] Robbart, M. L. , Aronson, R. B. , Deslarzes, K. J. , Precht, W. F. , Duncan, L. , Zimmer, B. , & DeMunda, T. (2009). Post‐hurricane assessment of sensitive habitats of the Flower Garden Banks vicinity. https://www.boem.gov/sites/default/files/boemnewsroom/Library/Publications/2009/25th_ITM_Source_Slide_Shows/3B_04_Deis_slide_show.pdf

[ece36340-bib-0060] Rocha, L. A. , Pinheiro, H. T. , Shepherd, B. , Papastamatiou, Y. P. , Luiz, O. J. , Pyle, R. L. , & Bongaerts, P. (2018). Mesophotic coral ecosystems are threatened and ecologically distinct from shallow water reefs. Science, 361(6399), 281–284. 30026226 10.1126/science.aaq1614

[ece36340-bib-0061] Serrano, X. , Baums, I. , O'Reilly, K. , Smith, T. , Jones, R. , Shearer, T. , … Baker, A. (2014). Geographic differences in vertical connectivity in the Caribbean coral *Montastraea cavernosa* despite high levels of horizontal connectivity at shallow depths. Molecular Ecology, 23(17), 4226–4240. 25039722 10.1111/mec.12861

[ece36340-bib-0062] Serrano, X. M. , Baums, I. B. , Smith, T. B. , Jones, R. J. , Shearer, T. L. , & Baker, A. C. (2016). Long distance dispersal and vertical gene flow in the Caribbean brooding coral *Porites astreoides* . Scientific Reports, 6(1), 1‐12. 10.1038/srep21619 26899614 PMC4761953

[ece36340-bib-0063] Shi, W. , Ayub, Q. , Vermeulen, M. , Shao, R.‐G. , Zuniga, S. , van der Gaag, K. , … Tyler‐Smith, C. (2010). A worldwide survey of human male demographic history based on Y‐SNP and Y‐STR data from the HGDP–CEPH populations. Molecular Biology and Evolution, 27(2), 385–393. 10.1093/molbev/msp243 19822636 PMC2806244

[ece36340-bib-0064] Shlesinger, T. , Grinblat, M. , Rapuano, H. , Amit, T. , & Loya, Y. (2018). Can mesophotic reefs replenish shallow reefs? Reduced coral reproductive performance casts a doubt. Ecology, 99(2), 421–437. 10.1002/ecy.2098 29205289

[ece36340-bib-0065] Smith, T. B. , Gyory, J. , Brandt, M. E. , Miller, W. J. , Jossart, J. , & Nemeth, R. S. (2016). Caribbean mesophotic coral ecosystems are unlikely climate change refugia. Global Change Biology, 22(8), 2756–2765. 10.1111/gcb.13175 26648385

[ece36340-bib-0066] Smith, T. B. , Holstein, D. M. , & Ennis, R. S. (2019). Disturbance in mesophotic coral ecosystems and linkages to conservation and management In Loya Y., Puglise K. A., & Bridge T. C. L. (Eds.), Mesophotic coral ecosystems (pp. 911–929). Berlin, Germany: Springer.

[ece36340-bib-0067] Sponaugle, S. , & Cowen, R. K. (2019). Coral ecosystem connectivity between Pulley Ridge and the Florida Keys In Loya Y., Puglise K. A., & Bridge T. C. L. (Eds.), Mesophotic coral ecosystems (pp. 897–907). Berlin, Germany: Springer.

[ece36340-bib-0068] Sponaugle, S. , Lee, T. , Kourafalou, V. , & Pinkard, D. (2005). Florida Current frontal eddies and the settlement of coral reef fishes. Limnology and Oceanography, 50(4), 1033–1048. 10.4319/lo.2005.50.4.1033

[ece36340-bib-0069] Staaterman, E. , Paris, C. B. , & Helgers, J. (2012). Orientation behavior in fish larvae: A missing piece to Hjort's critical period hypothesis. Journal of Theoretical Biology, 304, 188–196. 10.1016/j.jtbi.2012.03.016 22465113

[ece36340-bib-0070] Studivan, M. , & Voss, J. (2018). Population connectivity among shallow and mesophotic *Montastraea cavernosa* corals in the Gulf of Mexico identifies potential for refugia. Coral Reefs, 37(4), 1183–1196.

[ece36340-bib-0071] Szmant, A. M. (1991). Sexual reproduction by the Caribbean reef corals *Montastrea annularis* and *M. cavernosa* . Marine Ecology Progress Series, 74, 13–25. 10.3354/meps074013

[ece36340-bib-0072] Van Hooidonk, R. , Maynard, J. , & Planes, S. (2013). Temporary refugia for coral reefs in a warming world. Nature Climate Change, 3(5), 508–511. 10.1038/nclimate1829

[ece36340-bib-0073] van Oppen, M. , Bongaerts, P. , Underwood, J. N. , Peplow, L. M. , & Cooper, T. F. (2011). The role of deep reefs in shallow reef recovery: An assessment of vertical connectivity in a brooding coral from west and east Australia. Molecular Ecology, 20(8), 1647–1660. 10.1111/j.1365-294X.2011.05050.x 21410573

[ece36340-bib-0074] Vaz, A. C. , Paris, C. B. , Olascoaga, M. J. , Kourafalou, V. H. , Kang, H. , & Reed, J. K. (2016). The perfect storm: Match‐mismatch of bio‐physical events drives larval reef fish connectivity between Pulley Ridge mesophotic reef and the Florida Keys. Continental Shelf Research, 125, 136–146. 10.1016/j.csr.2016.06.012

[ece36340-bib-0075] Woodley, J. , Chornesky, E. , Cliffo, P. , Jackson, J. , Kaufman, L. , Knowlton, N. , … Rooney, M. (1981). Hurricane Allen's impact on a Jamaican coral reef. Science, 214, 13. 17744383 10.1126/science.214.4522.749

[ece36340-bib-0076] Wyers, S. , Barnes, H. , & Smith, S. (1991). Spawning of hermatypic corals in Bermuda: A pilot study In Williams R. B., Cornelius P. F. S., Hughes R. G., Robson E. A.(Eds,). Coelenterate biology: Recent research on Cnidaria and Ctenophora (pp. 109–116). Berlin, Germany: Springer.

[ece36340-bib-0077] Yund, P. O. (2000). How severe is sperm limitation in natural populations of marine free‐spawners? Trends in Ecology & Evolution, 15(1), 10–13. 10.1016/S0169-5347(99)01744-9 10603497

[ece36340-bib-0078] Zheng, X. , Levine, D. , Shen, J. , Gogarten, S. M. , Laurie, C. , & Weir, B. S. (2012). A high‐performance computing toolset for relatedness and principal component analysis of SNP data. Bioinformatics, 28(24), 3326–3328. 10.1093/bioinformatics/bts606 23060615 PMC3519454

