# Supplementary figures and images for: Fine‐scale structure among mesophotic populations of the great star coral Montastraea cavernosa revealed by SNP genotyping
Source: Ecol Evol. 2020 May 20;10(12):6009–19. doi: 10.1002/ece3.6340 (PMC7319168; doi:10.1002/ece3.6340)

Proportion of Reads Aligned

■ *Symbiodinium* ■ *Breviolum* ■ *Cladocopium* ■ *Durusdinium*

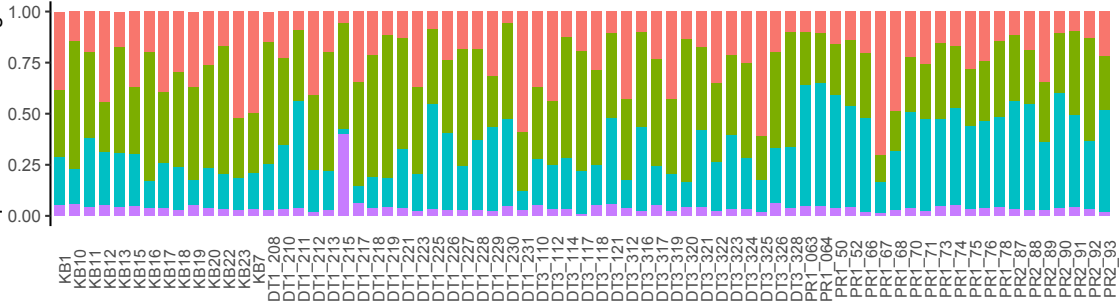

Supplement: Supplementary file 1 — Fig S1 [file ECE3-10-6009-s001.pdf]
